# Population-level transcriptomic datasets from two benthic invertebrates exposed to long-term experimental warming and acidification

**DOI:** 10.3897/BDJ.14.e186927

**Published:** 2026-05-11

**Authors:** Elisavet Kaitetzidou, Harris Markomanolaki, Francesco Fabiano, Carlotta Paone, Eva Chatzinikolaou, Thanos Dailianis

**Affiliations:** 1 Hellenic Centre for Marine Research, Institute of Marine Biology, Biotechnology and Aquaculture, Heraklion, Greece Hellenic Centre for Marine Research, Institute of Marine Biology, Biotechnology and Aquaculture Heraklion Greece; 2 School of Biology, Aristotle University of Thessaloniki, Thessaloniki, Greece School of Biology, Aristotle University of Thessaloniki Thessaloniki Greece; 3 Sicily Marine Centre, Stazione Zoologica Anton Dohrn, Sicily Marine Centre, Messina, Italy Sicily Marine Centre, Stazione Zoologica Anton Dohrn, Sicily Marine Centre Messina Italy; 4 University of Algavre, Faculty of Sciences and Technology, Faro, Portugal University of Algavre, Faculty of Sciences and Technology Faro Portugal

**Keywords:** climate change, Porifera, marine gastropods, mRNA sequencing, transcriptome, RNA-seq, environmental stressors, Mediterranean

## Abstract

Ocean warming and acidification are major drivers of change in marine ecosystems, with particularly strong impacts on low-mobility benthic organisms. Despite their ecological importance, genomic and transcriptomic resources from sponges (Phylum, Porifera) and marine gastropods (Phylum, Mollusca) that capture responses to long-term, combined climate stressors and population-level variability remain limited. Herein, we present population-level RNA-seq datasets from the sponge *Chondrilla nucula* and the gastropod *Hexaplex trunculus*, collected from northern and southern Aegean Sea (eastern Mediterranean) populations and exposed for three months to elevated temperature and reduced pH in a common-garden experiment simulating near-future climate change conditions. The datasets comprise high-quality paired-end Illumina reads, a complete *de novo* transcriptome assembly for *C. nucula* and genome-guided alignments for *H. trunculus*. These datasets provide a valuable resource for investigating transcriptional plasticity and climate change resilience in benthic marine invertebrates.

## Introduction

Climate change is altering marine environments through increasing seawater temperatures and declining pH (Bindoff et al. 2022) with significant consequences for marine biodiversity and ecosystem functioning. The altered conditions affect many marine organisms, whereas previous studies indicate that some groups face stronger negative effects than others (Kroeker et al. 2013, Lefevre 2016). Therefore, climate change is expected to increase physiological stress and disease susceptibility in most marine organisms (Marcogliese 2008), with corals exhibiting reduced calcification (Gazeau et al. 2007), increased bleaching events (Hughes et al. 2017) and decreased larval recruitment (Doropoulos et al. 2012) and molluscs showing impaired shell formation (Chatzinikolaou et al. 2017, Leung et al. 2017). Notably, sessile and low-mobility organisms are particularly vulnerable to these changes due to their limited ability to avoid the unfavourable conditions, making them valuable organisms for assessing the biological impacts of ocean warming and acidification.

Amongst such organisms, sponges and marine gastropods have key ecological roles in marine ecosystems, contributing to nutrient cycling, habitat structuring and trophic interactions (Coleman et al. 2006, Taylor et al. 2007, Bell 2008, Goeij JM et al. 2013, Coen and Bishop 2015, Barrientos-Lujan et al. 2019, Zhou et al. 2024). Despite their broad distribution and ecological importance, transcriptomic resources for these taxa remain limited, particularly with respect to data that capture population-level variability and responses to combined and long-term climate stressors (Thangaraj and Sun 2025). Most available molecular studies focus on short-term exposures (typically lasting less than a week), single stressors or single populations, limiting our understanding of transcriptional plasticity and potential local acclimatisation or adaptation (Gleason and Burton 2015, Kaniewska et al. 2015, Liu et al. 2025, Guzman and Conaco 2016, Tripp-Valdez et al. 2019, Koutsouveli et al. 2020).

Advances in high-throughput sequencing technologies now enable the generation of diverse and extremely informative molecular datasets (Shendure and Ji 2008, Melzner et al. 2026), facilitating studies aiming at the characterisation of organismal responses, including non-model species, under controlled experimental scenarios. RNA sequencing (RNA-seq), in particular, provides a powerful and unbiased approach for profiling genome-wide gene expression and is especially valuable for investigating stress responses in organisms lacking reference genomes (Ekblom and Galindo 2011).

Here, we present population-level RNA-seq datasets from two benthic invertebrates, the sponge *Chondrilla nucula* and the gastropod *Hexaplex trunculus*, derived from northern and southern Aegean Sea populations and exposed for three months to combined elevated temperature and reduced pH in a common-garden experiment simulating near-future climate change conditions. These datasets provide a rare resource for exploring transcriptional plasticity, population differentiation in stress responses and comparative molecular pathways across distant invertebrate phyla under long-term environmental stress.

### Value of the dataset

The datasets presented here comprise population-level RNA-seq resources from two benthic invertebrates, the sponge *C. nucula* and the gastropod *H. trunculus*, collected from northern and southern Aegean Sea populations and experimentally exposed for three months to combined elevated temperature and reduced pH, in a common-garden experiment simulating near-future climate change conditions.

These datasets are particularly valuable because they integrate long-term exposure, combined climate stressors and intraspecies variation within the same experimental framework, a design that remains rare in marine transcriptomic studies (Christou et al. 2025). By including populations distributed along an environmental gradient, the data enable investigation of intraspecific variation in baseline gene expression and in transcriptional responses to environmental stress, providing opportunities to explore mechanisms of transcriptional plasticity, local acclimatisation and potential adaptation.

In particular, intraspecific variation in stress responses is recognised as a critical component of species resilience to climate change (Nicastro et al. 2023) and populations distributed along environmental gradients may differ in their baseline gene expression profiles and in their transcriptional responses to environmental perturbations, reflecting local acclimatisation or adaptation (Schoville et al. 2012). Therefore, the datasets of this study were generated under controlled environmental stress experiments and constitute valuable resources for identifying molecular pathways involved in stress responses, elucidating transcriptional plasticity and enabling comparative analyses across species as well as amongst populations.

## Methods

In brief, the methodology involved two benthic species maintained in a common-garden experiment designed to simulate climate change scenarios. RNA-seq datasets were generated using Illumina high-throughput sequencing. Raw sequencing reads were processed using standard bioinformatic workflows for data quality assessment.

### Overall experimental framework

The RNA-seq datasets presented here were generated as part of a broader, integrative investigation which examined the responses of two invertebrate species to climate change at multiple levels of biological organisation — including morphological, physiological, transcriptomic and symbiotic community responses. The full description of the experimental design, mesocosm setup and organism sourcing and maintenance is provided in the companion article by Chatzinikolaou et al. (2026), part of the present article collection and currently available as a preprint. A summary of specimen collection and experimental treatment prior to RNA extraction and sequencing is provided thereafter.

### Specimen acquisition and experimental treatment

Specimens of *C. nucula* and *H. trunculus* were collected from two wild populations in the Aegean Sea: Crete, South Aegean (35.3357°N, 25.2815°E, ≤ 5 m depth) and Chalkidiki, North Aegean (39.9315°N, 23.7348°E, ≤ 5 m depth). Individuals were transferred to experimental mesocosms and maintained for three months under controlled conditions simulating present-day and projected climate scenarios.

Following an initial acclimatisation period at ambient 24ºC and pH = 8.1, three experimental scenarios were applied:

(i) Control: Ambient summer temperature set to 27°C (average of maximum summer temperatures recorded at both locations) and ambient pH (~ 8.1).

(ii) South Aegean Climate Change (SACC, “extreme”): Temperature set to 31°C (maximum summer temperature recorded in Crete +4°C) and pH reduced by 0.3 units (~ 7.8).

(iii) North Aegean Climate Change (NACC, “mild”): Temperature set to 30°C (maximum summer temperature recorded in Chalkidiki +4°C) and pH reduced by 0.3 units (~ 7.8).

The selected temperature increase (+4°C) and pH decrease (−0.3 units) follow projections under the high greenhouse gas emissions scenario (RCP 8.5) reported in the IPCC Climate Change 2023 Synthesis Report (Calvin et al. 2023).

Detailed descriptions of sampling, aquarium transfer, tank setup and organism maintenance are provided by Chatzinikolaou et al. (2026), part of the present article collection and currently available as a preprint. The present paper summarises only the procedures relevant to RNA extraction and sequencing. At the end of the experimental period, after three months of exposure in a common-garden experiment, four specimens from each experimental condition were collected, immediately frozen in liquid nitrogen and stored at −80°C prior to downstream analyses (Table 1).

### Sample processing

#### RNA extraction

**Extraction protocol setup**

To obtain RNA of sufficient quality and quantity for subsequent steps, a protocol designed for plant tissues and suggested by Jordon‐Thaden et al. (2015) was employed with minor alterations. This protocol combines CTAB (cetyltrimethylammonium bromide) extraction with an all-in-one commercial extraction solution which, in our case, was NucleoZOL, (Macherey-Nagel, Düren, Germany). In more detail, to isolate total RNA from the sponge tissue and the foot of the gastropod, each sample was first pulverised and lyophilised using a mortar and pestle in the presence of liquid nitrogen, thereby limiting RNA degradation. The CTAB extraction buffer was then added to the lysate, vortexed and incubated at 55°C to allow the dissociation of nucleoprotein complexes. Following the standard CTAB protocol, samples were centrifuged to separate the upper aqueous phase from the lower organic and interphase. The aqueous phase was transferred into a clean tube, mixed with a chloroform-isoamyl alcohol mixture and centrifuged. The aqueous phase was then mixed with NucleoZOL and sarcosyl, centrifuged and again transferred into clean tubes. The subsequent steps followed the NucleoZOL standard procedure. Total RNA was precipitated with isopropanol and the pellet was washed three times with 75% ethanol. The RNA pellet was then re-dissolved in nuclease-free water to a concentration of approximately 1 μg/μl and stored at -20°C overnight to facilitate optimal RNA self-hybridisation. Additional modification for both sponge and gastropod samples, included, Proteinase K treatment (10 mg/ml, 15 min at 55^o^C) and DNase treatment step targeting the complete elimination of DNA.

**Quality control of the isolated RNA**

RNA concentration and purity were determined spectrophotometrically using the NanoDrop 1000 (Thermo Fisher Scientific) by measuring the optical density and calculating the ratios: 260/280 nm and 260/230 nm. The integrity of the extracted RNA was qualitatively assessed via agarose gel electrophoresis using a 1.5% agarose gel (Sigma-Aldrich, Germany). Moreover, prior to library preparation, RNA integrity was re-assessed by Novogene where the samples were sent for library preparation and sequencing, using the TapeStation system (Agilent Technologies,, Santa Clara, USA). High-quality RNA was isolated from 24 *C. nucula* and 24 *H. trunculus* samples. RNA integrity and quality were assessed using agarose gel electrophoresis and TapeStation analysis, as shown in Fig. 1. High-quality RNA is critical for reliable transcriptomic analyses, as degraded samples can bias libraries and reduce sequencing efficiency. In invertebrates, taxa-specific RNA behaviour may deviate from standard patterns; for example, in gastropods, the 28S rRNA is often unstable, resulting in only the 18S band being visible (Barcia et al. 1997a), as observed in *H. trunculus* (Fig. 1c, d). Additionally, although RNA Integrity Numbers (RIN) were confirmed, the standard RIN ≥ 7 threshold was not strictly applied, since the extraction protocol was designed to retain both mRNA and miRNA for potential future analyses.

#### Library preparation and Sequencing

The construction of the libraries, as well as the sequencing, was performed by Novogene (Germany, Munich Sequencing Center Novogene GmbH). mRNA libraries were constructed with the directional mRNA library preparation kit suitable for sequencing with Illumina platforms. There were five key steps which included: i. Poly(A) selection of mRNA using oligo(dT) magnetic beads; ii. Fragmentation of the isolated mRNA and reverse transcription into cDNA using random primers; iii. End repair of the resulting cDNA fragments, adapter ligation and PCR amplification; iv. AMPure XP purification to remove primers, adapter dimers and undesired short fragments and v. Quality control of the constructed libraries using Qubit dsDNA HS assay and size distribution analysis using Agilent Bioanalyzer High Sensitivity DNA chips.

All constructed libraries exhibited concentrations within the expected range and total yields sufficient for sequencing. They met the quality standards required for high-throughput sequencing and were subsequently submitted for RNAseq.

Sequencing was performed in Novogene using the Illumina NovaSeq X Plus platform, generating 150 bp paired-end reads (PE150), targeting 6 Gb of sequencing data per sample for the sponge species and 9 Gb of sequencing data per sample for the gastropod species. Usually 6Gb of sequencing data is standard for transcriptomic analyses and provides sufficient depth and coverage for accurate quantification of gene expression. Although the genome of *C. nucula* has not yet been sequenced, related species within the same genus have an estimated genome size of 191 M. Therefore, the targeted 6 Gb of sequencing data per sample is more than sufficient for comprehensive transcriptome analysis. In contrast, *H. trunculus* is a gastropod with a substantially larger genome (~ 2.21 G) (Challis et al. 2023). For this species, 9 G of raw data per sample were generated to provide the necessary sequencing depth for robust downstream analyses.

### Data processing

Raw RNA-seq reads were passed through quality control, adapter removal and quality-based filtering. High-quality reads were then used to generate transcriptomic datasets with two different strategies: *de novo* transcriptome assembly for *C. nucula* and genome-guided alignment for *H. trunculus*. Further details of the data procesing are described in this section.

#### Quality control

Quality assessment of each sample was performed using FastQC v.0.11.9 (Andrews 2010) on the HCMR Zorba cluster (https://hpc.hcmr.gr/) (Zafeiropoulos et al. 2021). The resulting reports were aggregated and summarized across all samples for each species using MultiQC (Ewels et al. 2016a, Ewels et al. 2016b). All samples met quality standards, providing high-quality input for downstream analyses.

#### Filtering and trimming

Illumina paired-end reads were cleaned using Trimmomatic v.0.39 (Bolger et al. 2014) in paired-end mode on the HCMR Zorba cluster. Adapter sequences were removed using the provided Illumina adapter file, allowing up to three mismatches in the seed region. Low-quality bases were trimmed using a sliding window of four bases, discarding bases when the average quality in the window dropped below 14. Reads shorter than 36 bases after trimming were discarded. More than 96% of reads survived quality filtering in both R1 and R2 files. The number of reads retained after filtering and used for downstream analyses is reported in Tables 2, 3.

#### *De novo* assembly

**Figure 2.**
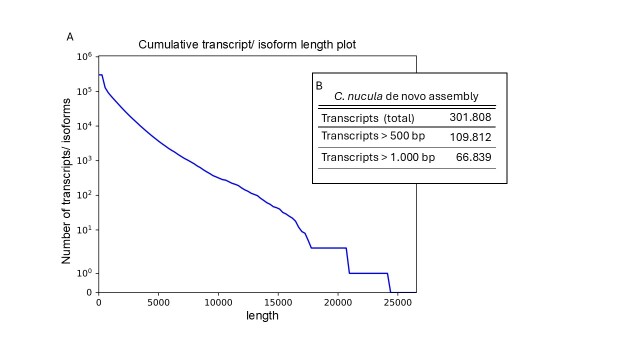
**(A)** Cumulative length distribution of transcripts (isoforms) in the *C. nucula de novo* assembly. The plot shows in log scale the number of assembled transcripts at different transcript lengths; **(B)** The total number of transcripts found in the assembly and the number of transcripts of more than 500 bp and more than 1000 bp length.

**Figure 3.**
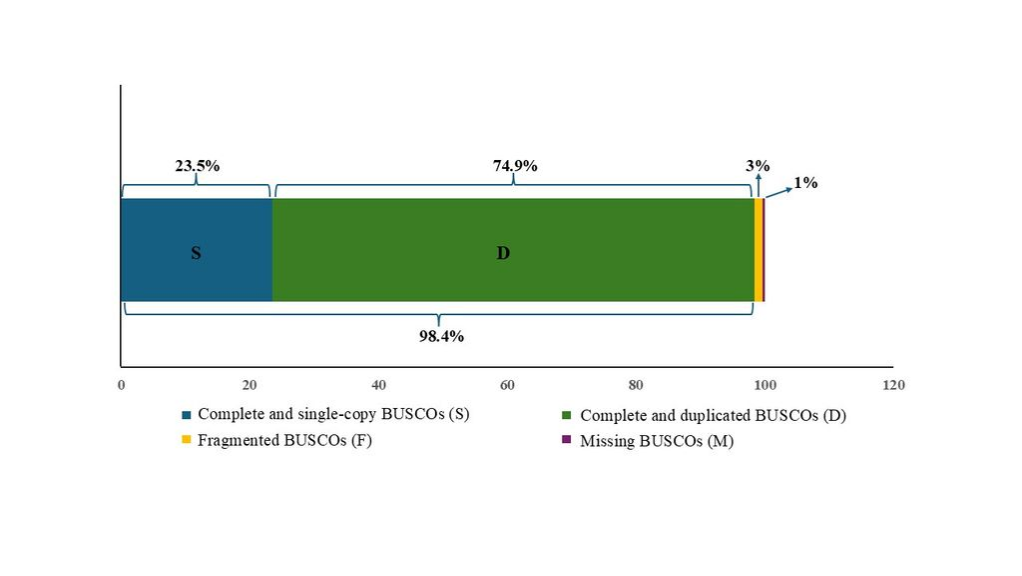
BUSCO assessment of the *C. nucula de novo* transcriptome assembly showing high completeness, based on the eukaryota_odb10 lineage dataset.

Since the genome of *C. nucula* is not available, a *de novo* reference transcriptome was assembled. Filtered reads were assembled using RNAspades (Bushmanova et al. 2019) (version: 4.2.0 (Galaxy platform, IUC toolshed) with paired-end reads, FR orientation, default K-mer detection and default Phred quality offset. The resulting assembly was used as a reference dataset for the downstream gene expression analysis for the sponge species. Assembly quality was evaluated using rnaQUAST (Bushmanova et al. 2016) (version: 2.3.0) (Galaxy, IUC toolshed) (Fig. 2). Assembly completeness was further assessed with BUSCO (Simão et al. 2015) (version: 5), using the eukaryota_odb10 lineage dataset (Creation date: 08-01-2024, number of genomes: 70, number of BUSCOs: 255). BUSCO analysis indicated a highly complete *de novo* transcriptome, with 98.4% of the expected eukaryotic lineage genes detected. Of these, 23.5% were identified as single-copy BUSCOs, while 74.9% were classified as duplicated (Fig. 3).

#### Genome-guided Alignment

Given the availability of a reference scaffold for *H. trunculus* (available on NBCI with the BioSampleID SAMN41141298), a genome-guided approach was utilised. Quality-filtered reads were aligned to the reference genome using HISAT3 (Zhang et al. 2021), optimised for spliced alignment. The mapping efficiency was exceptionally high and consistent, with alignment rates ranging from 81.2% to 97.3%. Most replicates exhibited a mapping rate of approximately 90%, confirming that the reference scaffold served as a robust framework for transcript quantification. The average percentage of total reads mapped to the genome is shown in Table 4.

## Biodiversity scope

The datasets, described in the present work, provide transcriptomic resources for two ecologically important benthic inverterates: the sponge *C. nucula* Schmidt 1862 (Porifera, Demospongiae) and the gastropod *H. trunculus* (Linnaeus, 1758) (Mollusca, Gastropoda), taxa for which genomic and transcriptomic resources remain comparatively limited. Both species are widespread in Mediterranean coastal ecosystems and contribute substantially to benthic habitat structure, nutrient cycling and trophic interactions.

Importantly, the data originate from populations distributed along a natural environmental gradient in the Aegean Sea and from individuals exposed for three months to combined warming and acidification in a controlled common-garden experiment. As such, the datasets are relevant for biodiversity research addressing how ecologically significant benthic taxa may respond to projected climate change conditions, enabling investigations of transcriptional plasticity, population differentiation in stress responses and comparative molecular pathways across distant invertebrate lineages.

### Target

Marine benthic invertebrates, including the sponge *Chondrilla nucula* and the gastropod *Hexaplex trunculus*.

### Taxonomic range

Kingdom Animalia; Phylum Porifera (*Chondrilla nucula*) and Phylum Mollusca (*Hexaplex trunculus*).

### Functional range

Transcriptomic datasets, capturing transcriptome-wide gene expression under controlled exposure to elevated temperature and reduced pH, while providing insights on the intraspecifics vatiation.

## Data Resources

Raw sequencing data generated by RNA sequencing are available in the NCBI Sequence Read Archive (SRA), under BioProject **PRJNA1413336** for *C. nucula* and BioProject **PRJNA1412568** for *H. trunculus*. Each BioProject includes 24 BioSamples, with all corresponding SRA experiments and runs linked, providing full access to the raw data for both species. The BioSamples connected to BioProject PRJNA1413336 are SAMN54875027 to SAMN54875050 (Suppl. material 1) and the BioSamples connected to BioProject PRJNA1412568 are SAMN54870970 and SAMN54871159 to SAMN54871181 (Suppl. material 2). All associated SRA runs can be assessed via the corresponding BioProject links.

### Resource 1

Download URL: https://www.ncbi.nlm.nih.gov/bioproject/?term=PRJNA1413336

Resource identifier: RNA-seq raw reads of *Hexaplex trunculus* under simulated climate change conditions

Data format : FASTQ

### Resource 2

Download URL: https://www.ncbi.nlm.nih.gov/bioproject/?term=PRJNA1412568 

Resource identifier: BioProject: PRJNA1412568, BioSamples: SAMN54870970 and SAMN54871159 to SAMN54871181

Data format : FASTQ

## Supplementary Material

75122245-3E75-5942-BBDF-6D2C85406AF2 10.3897/BDJ.14.e186927.suppl1 Supplementary material 1 Population-level transcriptomic datasets from two benthic invertebrates exposed to long-term experimental warming and acidification Data type Sequencing metadata file Brief description Supplementary Table S1. Metadata associated with RNA sequencing of *Chondrilla nucula* samples. The table includes detailed information for each sample, including sample identifiers, corresponding BioSample and SRA run accessions, collection details (location, coordinates, date) and sequencing information (library preparation method, sequencing platform, read type). The metadata are derived from the BioSample and SRA experiment records associated with the corresponding NCBI BioProject. File: oo_1589011.tsv https://binary.pensoft.net/file/1589011 Elisavet Kaitetzidou, Harris Markomanolaki, Francesco Fabiano, Carlotta Paone, Eva Chatzinikolaou, Thanos Dailianis

61098945-7115-54A4-9196-0FFC38C5B522 10.3897/BDJ.14.e186927.suppl2 Supplementary material 2 Population-level transcriptomic datasets from two benthic invertebrates exposed to long-term experimental warming and acidification Data type Sequencing metadata file Brief description Supplementary Table S1. Metadata associated with RNA sequencing of *Hexaplex trunculus* samples. The table includes detailed information for each sample, including sample identifiers, corresponding BioSample and SRA run accessions, collection details (location, coordinates, date) and sequencing information (library preparation method, sequencing platform, read type). The metadata are derived from the BioSample and SRA experiment records associated with the corresponding NCBI BioProject. File: oo_1589034.tsv https://binary.pensoft.net/file/1589034 Elisavet Kaitetzidou, Harris Markomanolaki, Francesco Fabiano, Carlotta Paone, Eva Chatzinikolaou, Thanos Dailianis

## Figures and Tables

**Figure 1a.**
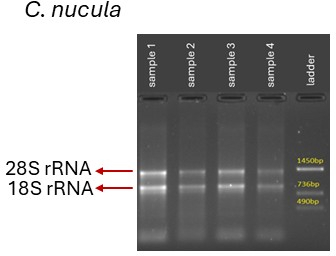
*C. nucula* RNA visualised by agarose gel electrophoresis;

**Figure 1b.**
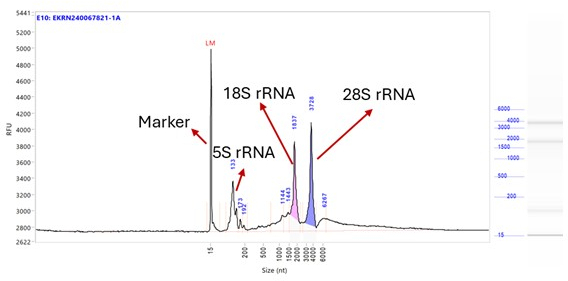
*C. nucula* RNA analysed using TapeStation;

**Figure 1c.**
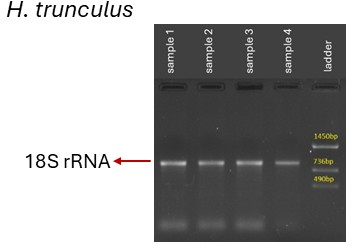
*H. trunculus* RNA visualised by agarose gel electrophoresis;

**Figure 1d.**
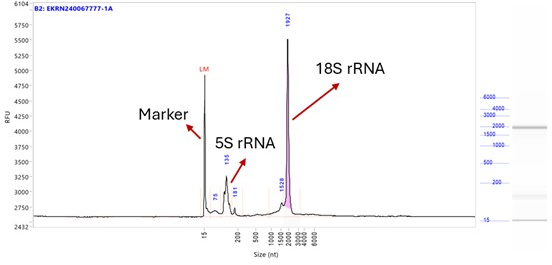
*H. trunculus* RNA analysed using TapeStation.

**Table 1.** Number of specimens per experimental condition used for gene expression analysis.

Species/ Population	Control	North Aegean Climate Change scenario	South Aegean Climate Change scenario
*C. nucula*/ North population	4 specimens	4 specimens	4 specimens
*C. nucula*/ South population	4 specimens	4 specimens	4 specimens
*H. trunculus*/ North population	4 specimens	4 specimens	4 specimens
*H. trunculus*/ South population	4 specimens	4 specimens	4 specimens

**Table 2.** Average number of sequencing reads and filtered reads per experimental condition for *C. nucula*.

Condition	Population	Average* number of Sequencing reads (Million reads) *(4 biological replicates)	Average* number of filtered reads (Million reads) *(4 biological replicates)
Control	N	21.57	20.75
North Aegean Climate Change scenario	N	28.77	27.36
South Aegean Climate Change scenario	N	19.70	19.01
Control	S	24.00	23.14
North Aegean Climate Change scenario	S	21.04	20.32
South Aegean Climate Change scenario	S	21.94	21.04

**Table 3.** Average number of sequencing reads and filtered reads per experimental condition for *H. trunculus*.

Condition	Population	Average* number of sequencing reads (Million reads) *(4 biological replicates)	Average* number of filtered reads (Million reads) *(4 biological replicates)
Control	N	33.43	32.07
North Aegean Climate Change scenario	N	60.32	58.98
South Aegean Climate Change scenario	N	61.38	56.65
Control	S	39.67	37.75
North Aegean Climate Change scenario	S	53.59	51.71
South Aegean Climate Change scenario	S	51.25	48.69

**Table 4.** Average percentage of total reads, aligned to the reference genome per condition.

Condition	Population	Average* percentage of reads mapped to the genome *(4 biological replicates)
Control	N	93.33%
North Aegean Climate Change scenario	N	85.1%
South Aegean Climate Change scenario	N	89.4%
Control	S	90.3%
North Aegean Climate Change scenario	S	89.35%
South Aegean Climate Change scenario	S	88.28%
